# Spatiotemporal Pavlovian head-fixed reversal learning task for mice

**DOI:** 10.1186/s13041-022-00952-5

**Published:** 2022-09-07

**Authors:** Kohei Yamamoto, Kota Yamada, Saya Yatagai, Yusuke Ujihara, Koji Toda

**Affiliations:** 1 Department of Psychology, Keio University, Mita 2-15-45, Minato-ku, Tokyo, 108-8345 Japan; 2 Japan Society for Promotion of Science, Chiyoda-ku, Tokyo, Japan; 3 Department of Anatomy and Neurobiology, University of Tennessee Health Science Center, Memphis, TN USA

**Keywords:** Reversal learning, Behavioral flexibility, Head-fixed, Pavlovian conditioning, Mice

## Abstract

Our world is full of uncertainty. Animals, including humans, need to behave flexibly to adjust to ever-changing environments. Reversal learning tasks have been used to assess behavioral flexibility in many species. However, there are some limitations in the traditional free-moving methodology, including (1) sessions to train the animals, (2) within-session number of trials associated with reversals, (3) factors of physical movement unrelated to the task in the maze or operant box, and (4) incompatibility with techniques, such as two-photon imaging. Therefore, to address these limitations, we established a novel spatiotemporal Pavlovian head-fixed reversal learning task for mice. Six experimentally naive adult C57BL/6J mice were used in this study. First, we trained head-fixed mice on a fixed-time schedule task. Sucrose solution was delivered every 10 s with a single drinking spout placed within the licking distance of the mice. After the mice showed anticipatory licking toward the timing of sucrose solution delivery, we began training the mice on the fixed-time schedule reversal learning task with two licking spouts. In this task, sucrose solution was delivered through one of the two drinking spouts. The rewarding spout was switched every 10 trials. Mice quickly learned to switch anticipatory licking to the rewarding side of the spouts, suggesting that they learned this head-fixed reversal learning task. Using the head-fixed experimental design, behavioral measures can be simplified by eliminating the complex behavioral sequences observed in free-moving animals. This novel head-fixed reversal learning task is a useful assay for studying the neurobiological mechanism of behavioral flexibility that is impaired in various psychopathological conditions.

## Introduction

Since our world is full of uncertainty, safe places may become dangerous and abundant resources may be exhausted. If environmental conditions fluctuate, animals, including humans, need to move to a new, more suitable environment. Flexible switching of behavior is essential for survival in ever-changing environments.

Reversal learning tasks have been widely used across species to investigate their ability to switch behaviors [1]. In the reversal learning task, two different stimuli are trained to predict two distinct outcomes. Thereafter, the contingency between the stimuli and outcomes is set to be reversed once or multiple times during the task. In this reversal learning task, organisms need to detect that the outcome is different from the prediction, inhibit the previously learned behavior, learn the reversed new stimulus-outcome contingencies, and maintain the new contingencies until the next reversal. Therefore, the reversal learning task has been used to assess behavioral flexibility.

Reversal learning is impaired in various psychopathological conditions, such as substance abuse, obsessive compulsive disorder, psychopathy, Parkinson’s disease, and schizophrenia [1]. Neuroimaging and neuropsychological studies in humans and ablation, unit recording, and circuit manipulation studies in animals have repeatedly emphasized the importance of the orbitofrontal cortex (OFC), medial prefrontal cortex, amygdala, and striatum [1–4]. Despite extensive studies investigating the neurobiological mechanisms of reversal learning, the detailed underlying circuit is unclear.

Modified versions of reversal learning tasks, initially conceived for rats, have been developed for mice [5]. Numerous types of tasks with different sensory modalities and response requirements have been reported, such as learning with a maze, including the Morris water maze and T-maze [6] and eight-arm maze [7]; with a two-choice digging task [8–10]; and with operant learning equipment, including the go/no-go task [11, 12] and delayed non-match-to-position task [13] or visual discrimination paradigms [14–16]. Reversal learning tasks are not limited to operant conditioning tasks. Reversal learning tasks with Pavlovian conditioning have also been used to study the mechanisms of behavioral flexibility [17]. Accumulating evidence suggests that mice can inhibit previously learned behavior, learn reversed new stimulus–outcome contingencies, and maintain new contingencies until the next reversal in both Pavlovian and operant conditioning.

Using mice as subjects enables cutting-edge molecular biology techniques to manipulate and measure neuronal activity in specific circuits. Along with recent advancements in optogenetics, chemogenetics, and calcium imaging, the need for behavioral tasks that are more feasible for molecular biology technologies is increasing. In addition, recent advances in machine learning have allowed us to objectively measure the detailed structure of behavior [18–20]. However, there are some limitations in the traditional free-moving methodology: (1) it is time consuming to train animals on the reversal learning task with free-moving operant conditioning, (2) it is not possible to obtain the number of trials associated with many reversals, and (3) the factors of physical movement unrelated to the task in the maze or operant box are large. To address these limitations, we established a novel head-fixed Pavlovian reversal learning task for mice.

## Results

**Fig. 1:**
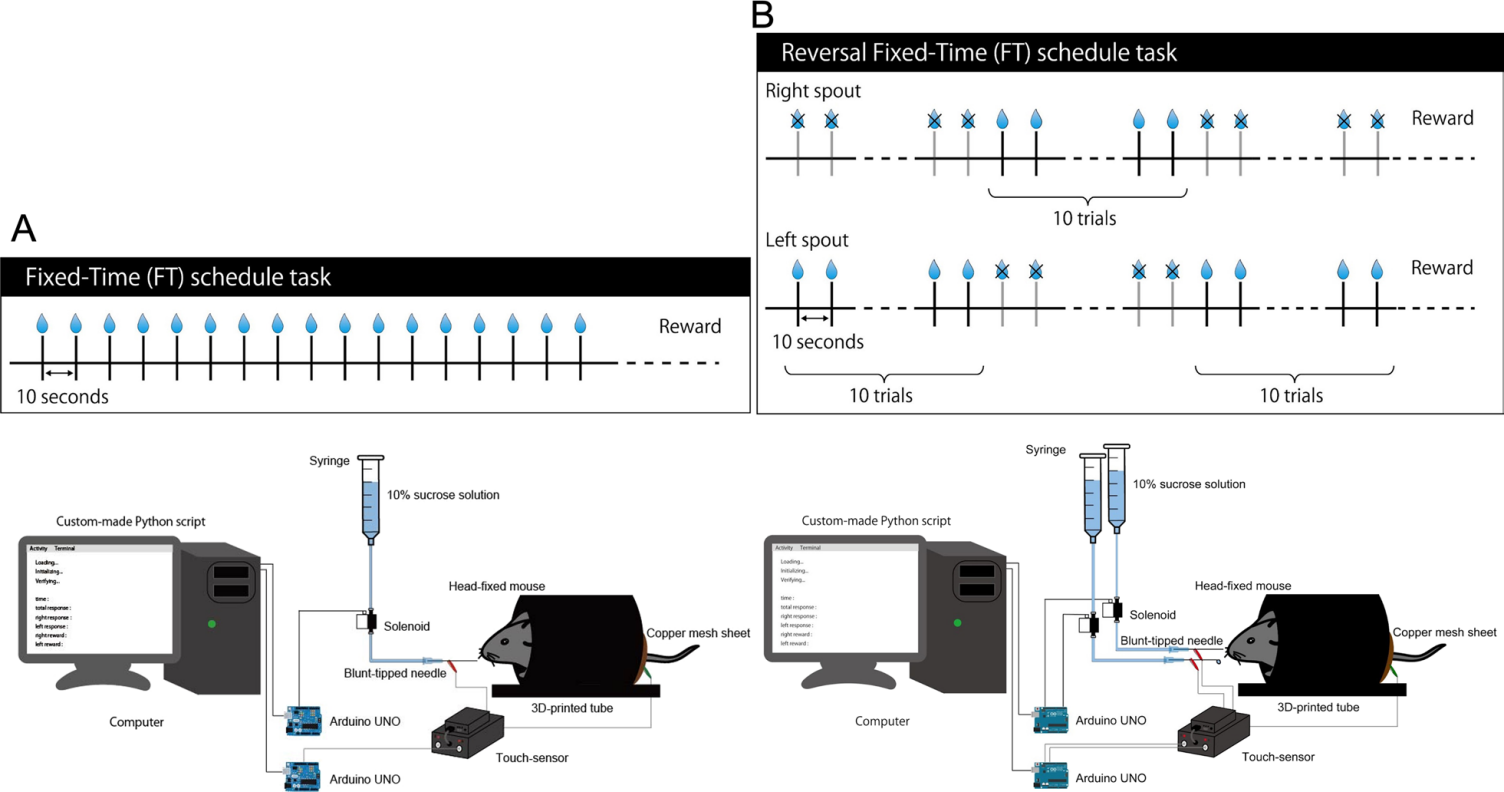
Experimental setup. **A** In the fixed-time schedule task, approximately 2 μL of 10% sucrose solution was delivered through the licking spout at 10 s interval. **B** The experimental setup of the fixed-time reversal learning task was exactly the same as that of the fixed-time schedule task, except that two drinking steel spouts were placed in front of the mouth of the animal. The distance between each licking spout was set at 4.5 mm. Sucrose solution was delivered through one of the two spouts. The rewarding spout was switched every 10 trials. The amount of sucrose solution delivered was calibrated to the same amount between each rewarding spout

**Fig. 2:**
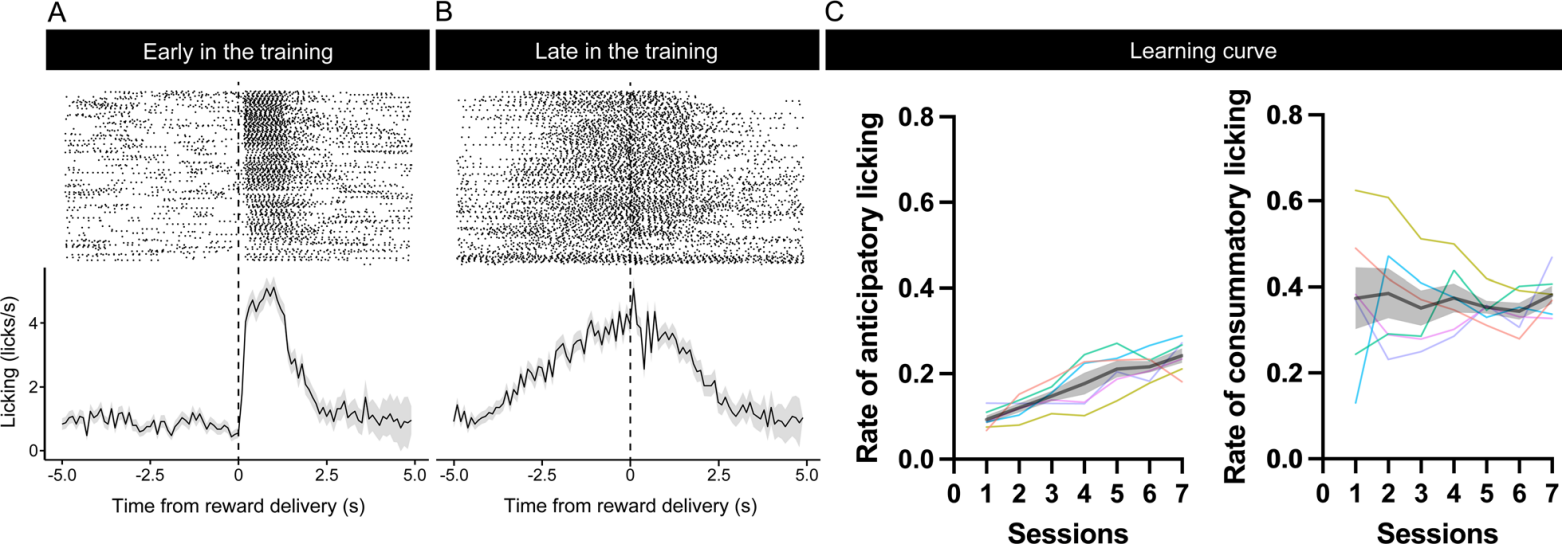
Comparison of performance in the early and late sessions of training and learning curve of the mouse on the fixed-time schedule task. Raster plot and histogram of licking aligned with the timing of sucrose solution delivery. We defined anticipatory licking as licking from 2 to 0 s before reward delivery and consummatory licking as licking from 0 to 2 s after reward delivery. **A** Raster plot (upper panel) and histogram (lower panel) of licking data in the early training session (session 2). At the beginning of training, mice did not show anticipatory licking but licked the spout after sucrose solution delivery. **B** Raster plot (upper panel) and histogram (lower panel) of licking data in the late training session (session 7). After training, mice showed anticipatory licking toward the timing of sucrose solution delivery. The frequency of anticipatory licking increased with an increase in the number of training sessions. **C** Learning curve of performance on fixed-time schedule task in all training sessions. The frequency of anticipatory licking increased with an increase in the number of training sessions (Left). The frequency of consummatory licking did not change with an increase in the number of training sessions (Right). Each color indicates the data for each individual subject

First, six mice were trained on a 10-s fixed-time schedule task (Fig. 1). Head-fixed mice licked a blunt-tipped drinking needle placed in front of their mouth, which delivered 10% sucrose solution every 10 s. At the beginning of training, mice did not show anticipatory licking, but licked the spout after sucrose solution delivery (Fig. 2A). After training, mice showed anticipatory licking toward the timing of sucrose solution delivery, suggesting that mice could predict the timing of reward delivery (Fig. 2B). The rate of anticipatory licking increased with the number of training sessions (Fig. 2C; *F* (2.065, 10.32) = 19.89, *p* = 0.0003, repeated-measure one-way ANOVA). The rate of consummatory licking did not change with the number of training sessions (Fig. 2D; *F* (2.294, 11.47) = 0.22, *p* = 0.8360, repeated-measure one-way ANOVA).

**Fig. 3:**
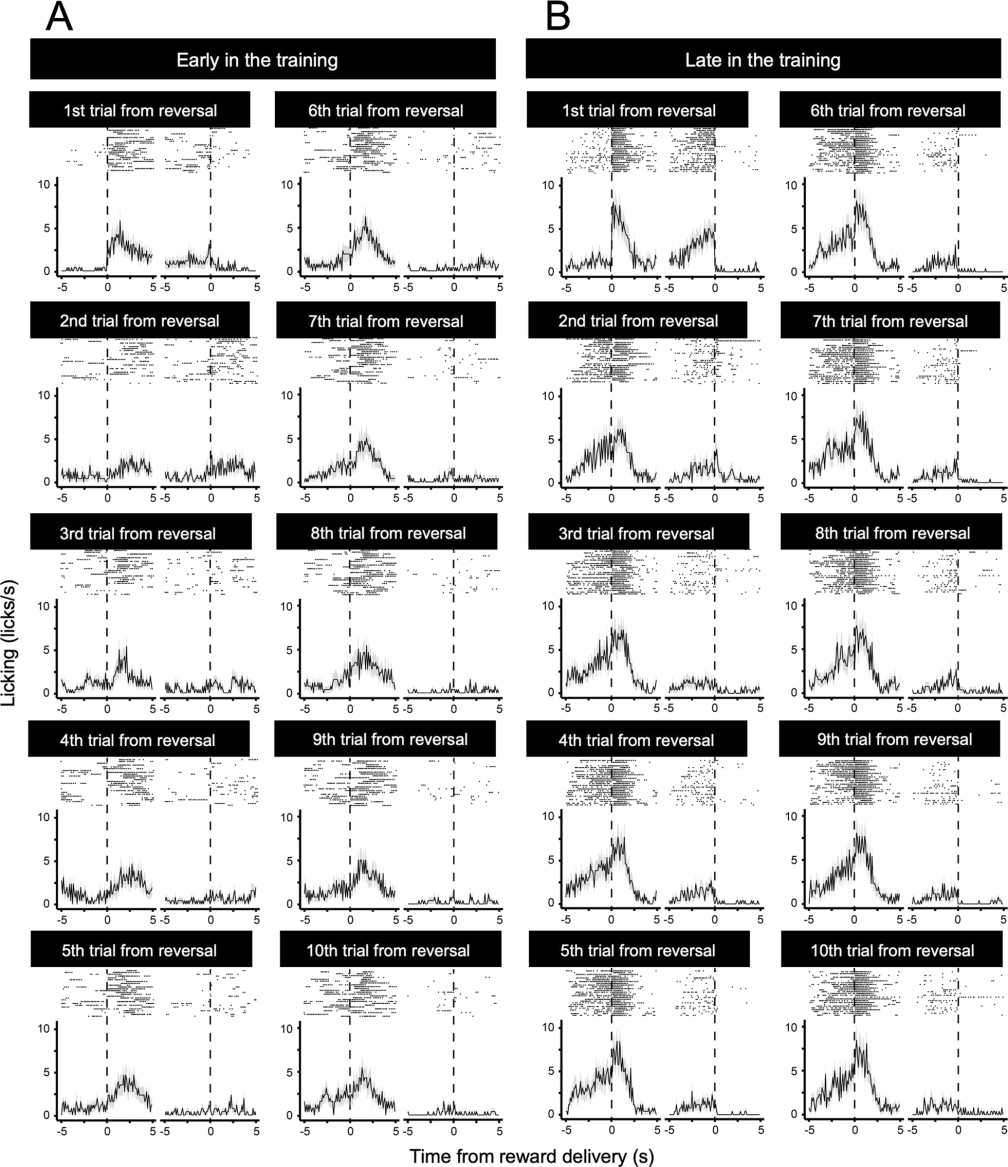
Example of the raster and histogram of correct and error responses in early and late sessions. **A**, **B** Horizontal axis indicates the time from the reward delivery. Vertical axis indicates the frequency of the licking. Upper panel indicates the raster plot. Lower panel indicates the histogram. In each panel, the raster and histogram of correct and error trials are shown on the left and right, respectively. Each condition consists of 25 trials. Example data of one mouse are shown

**Fig. 4:**
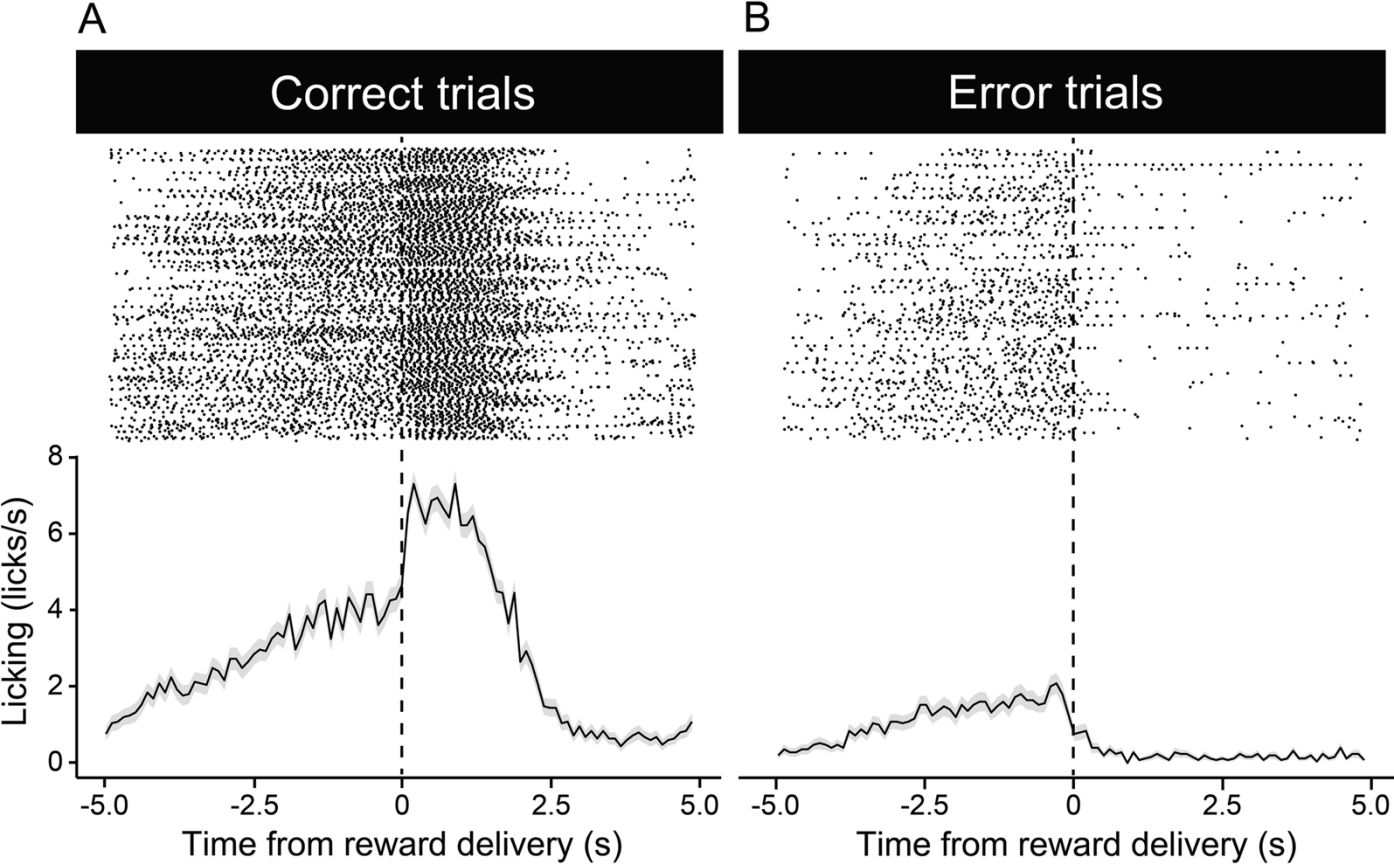
Example of the correct and error responses after training on the reversal learning task. **A** Example of the raster and histogram of a correct licking response. **B** Example of the raster and histogram of an error licking response. Horizontal axis indicates the time from the reward delivery. Vertical axis indicates the frequency of licking. Example data on the seventh day of training with 250 trials are shown. *N* = 250 (trials)

After mice showed anticipatory licking, we began to train the mice on the fixed-time schedule reversal learning task with two licking spouts (Fig. 1B). In this reversal learning task, we delivered sucrose solution through one of the two spouts. The rewarding spout was switched every 10 trials. There was no external cue to indicate the timing of the switching. We defined licking toward the rewarding side of the spout as a correct response and licking toward the other spout as an error response. Mice quickly learned to change anticipatory licking to the rewarding side spout after reversal. Figure 3 shows an example of the performance of a mouse on the reversal learning task in the early (Session 2) and late (Session 7) training sessions. In the early session (Session 2), the mouse showed sparse anticipatory licking of the rewarding spout in all trials (Fig. 3A). In contrast, in the late session (Session 7), the mouse showed anticipatory licking of the rewarding spout even in the trial immediately after reversal (Fig. 3B). After training, mice showed a build-up ramping pattern of licking until the timing of the reward delivery in both correct and incorrect trials (Fig. 4). These data suggested that mice could learn to flexibly change the prediction of (1) the timing of reward delivery and (2) location of the rewarding spout immediately after reversal.

**Fig. 5:**
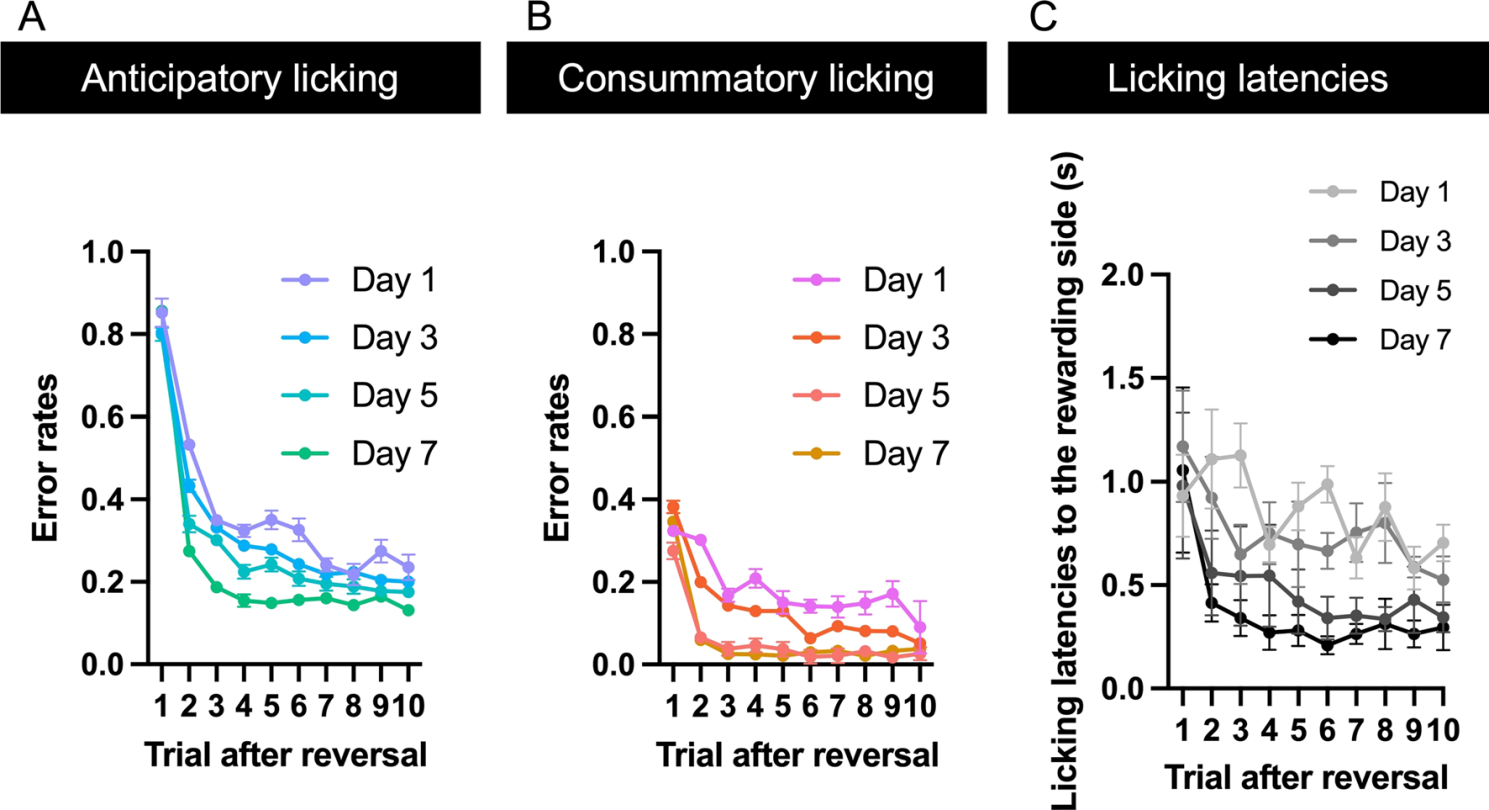
Overall relationship between error rates and trial after reversal. **A** Overall relationship between error rates and trials after reversal in anticipatory licking. **B** Overall relationship between error rates and trials after reversal in anticipatory licking. The horizontal axis indicates the number of trials after reversal. The vertical axis indicates the error rate. We defined the licking toward the rewarding side of the spout as a correct response and licking toward the non-rewarding spout as an errored response. **C** Licking latencies to the rewarding side of the spout. The horizontal axis indicates the number of trials after reversal. The vertical axis indicates the latencies of licking toward the rewarding side of the spout. Each color indicates the experimental day after the start of the training of the reversal learning task. Only the data collected on days 1, 3, 5, and 7 are shown. Because the experiment of one out of six mice was discontinued by the trouble of the head-plate, we could acquire the data of the mouse for four sessions. *N* = 6 (subjects)

**Fig. 6:**
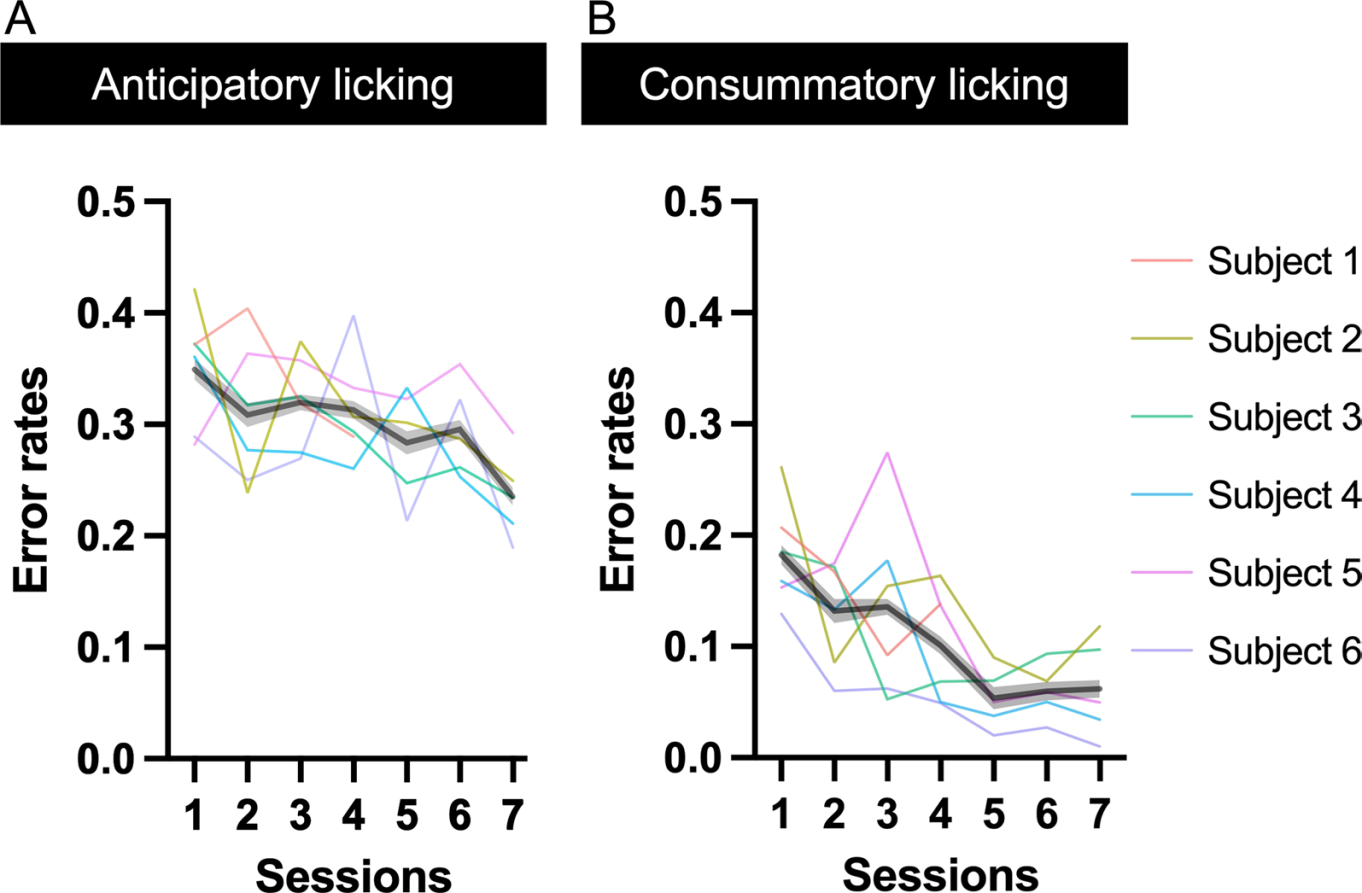
Overall learning curve of the reversal learning task. **A** Overall learning curve of anticipatory licking in the reversal learning task. **B** Overall learning curve of consummatory licking in the reversal learning task. The horizontal axis indicates the number of sessions after the start of training on the fixed-time reversal learning task. The vertical axis indicates the error rates of mice. We defined licking toward the rewarding side of the spout as a correct response and licking toward the non-rewarding spout as an error response. Different colors indicate the corresponding data for each individual mouse. Because the experiment of one out of six mice was discontinued by the trouble of the head-plate, we could acquire the data of the mouse for four sessions. *N* = 6 (subjects)

Figure 5 shows the relationship between error rates and trials after reversal in all mice. On the first day of the reversal learning task, the performance of three of six mice was very low because it was the first time that the mice licked both the right and left sides of the rewarding spouts. Therefore, we excluded three data points on Day 1 from the analysis. Although three of six mice took one day to learn to lick both the right and left sides of the rewarding spouts, all mice performed the task consistently on the second day of the reversal learning training. Because the experiment of one out of six mice was discontinued by the trouble of the head-plate, we could acquire the data of the mouse for four days. In all sessions, mice showed typical behavioral characteristics of reversal learning performance. The error rates increased after reversal and decreased as the trial progressed both in anticipatory (Fig. 5A; a significant main effect of “trials after the reversal”: *F* (9, 54) = 229.4, *p* < 0.0001, two-way ANOVA) and consummatory licking (Fig. 5B; a significant main effect of “trials after the reversal”: *F* (9, 54) = 65.45, *p* < 0.0001, two-way ANOVA). During training, the error rates gradually decreased in all trials both in anticipatory (Fig. 5A; a significant main effect of “days of training”: *F* (6, 54) = 15.52, *p* < 0.0001, two-way ANOVA) and consummatory licking (Figs. 5B; a significant main effect of “days of training”: *F* (6, 54) = 32.53, *p* < 0.0001, two-way ANOVA). The overall error rates gradually decreased with training both in anticipatory (Fig. 6A; *F* (6, 32) = 2.699, *p* = 0.03, one-way ANOVA) and consummatory licking (Fig. 6B; *F* (6, 32) = 4.957, *p* = 0.0011, one-way ANOVA). Overall, error rates of anticipatory licking were higher than those of consummatory licking. In consummatory licking, sucrose solution was delivered through the rewarding side of the spout; therefore, mice could switch the side to lick after the presentation of sucrose solution. Moreover, the latencies to licking the rewarding side of the spout were longer in the trial after reversal and decreased as the trial progressed (Fig. 5C; a significant main effect of “trials after the reversal”: *F* (9, 54) = 16.30, *p* < 0.0001, two-way ANOVA) and were gradually decreased along with the training (Fig. 5C; a significant main effect of “days of training”: *F* (6, 54) = 20.71, *p* < 0.0001, two-way ANOVA).

## Discussion

In this study, we established a novel spatiotemporal head-fixed Pavlovian reversal learning task for mice. In the fixed-time schedule task with a single licking spout, mice showed anticipatory licking toward the timing of reward delivery after training, suggesting that mice learned to predict the timing of the reward, as reported in our previous study [21]. After mice learned the fixed-time schedule task, we trained them on the fixed-time reversal learning task with two drinking spouts. After every 10 trials, the rewarding side was reversed in the reversal learning situation. Mice quickly switched the anticipatory licking to the rewarding side of the spouts, even on the first or second day of training. Errors in anticipatory and consummatory licking decreased during training. This result indicates that mice can learn this head-fixed Pavlovian reversal learning task quickly. Licking responses on this head-fixed Pavlovian reversal learning task can be accurately quantified and show the hallmarks of the results of the reversal learning task in free-moving operant conditioning. After reversal of the contingency between the stimuli and outcomes, a substantial error rate was observed, which decreased as the trials progressed.

The results obtained from the head-fixed reversal learning task showed the hallmarks of the results of the traditional reversal learning tasks. After reversal of the rewarding licking spout, high error rates were observed in both anticipatory and consummatory licking. The overall error rates decreased with training. Errors of consummatory licking were stable in the fifth session of the training, but errors of anticipatory licking were decreased from the fifth to seventh session of the training, suggesting that the effect of learning is relatively slower in anticipatory licking. This head-fixed procedure allows us to analyze (1) conditioned and unconditioned responses as anticipatory and consummatory licking, (2) acquisition and maintenance of the temporal prediction of the timing of the reward, (3) acquisition and maintenance of reversal learning, (4) errors soon after reversal, and (5) maintenance of the response after reversal. In particular, reversal learning and maintenance of reversed responses are dissociated in different brain areas. Chudamasa and Robbins [16] compared the effects of excitotoxic lesions of the OFC and infralimbic cortex (ILC) in the visual discrimination reversal learning task in rats. When the stimulus–reward contingencies were reversed, more errors were observed in both the OFC and ILC lesion groups, but only the OFC lesion group was unable to suppress the previously rewarded responses, committing more “stimulus perseverative” errors. In contrast, the ILC group showed a pattern of errors that were attributable to “learning” than perseveration. Although animals need to learn from the reward omission in the trial after reversal, they should maintain the newly learned stimulus–reward contingency throughout other trials. These two learning processes are distinct, suggesting that the corresponding neurobiological mechanisms are dissociated. Therefore, our spatiotemporal Pavlovian head-fixed reversal learning task could be a useful behavioral approach to uncover the psychological and neurobiological mechanisms of behavioral flexibility.

This spatiotemporal Pavlovian head-fixed reversal learning task can be extended in the future. One direction is to manipulate the task difficulty. The difficulty of this reversal learning task can be manipulated by changing the temporal interval between reward delivery. This manipulation may be important when considering the effect of working memory load on reversal learning performance. Another direction is the implementation of outcome probabilities. If the reversal learning task is not a probabilistic design, organisms can use a win–stay and lose–shift strategy. This can be simple discrimination learning triggered by extinction or outcome omission (IF there is no reward, THEN do another response). To exclude such a possibility, making the task probabilistic may be useful for studying behavioral flexibility in detail. The third direction of the extension is to invent the head-fixed Pavlovian set-shifting task based on the current reversal learning task. By adding visual, auditory, and/or other modalities, the current head-fixed Pavlovian spatiotemporal reversal learning task can be extended to the set-shifting task as a Wisconsin Card Sorting-like task. For example, the contingency between the stimuli and outcome may be based on visual, auditory, and spatial cues. As previously demonstrated, the head-fixed Pavlovian procedure can reduce the amount of training compared to free-moving operant conditioning [21]. Comparing the results of the head-fixed Pavlovian task with those of the existing head-fixed operant task may be important for understanding the psychological and neurobiological mechanisms of behavioral flexibility.

Recent technical advances in molecular biology and machine learning have allowed us to investigate the relationship between behavior and its neurobiological correlates. The behavioral task developed in this experiment can be combined with calcium imaging techniques. Because of the advantage of the head-fixed condition, the location of the brain is constant while the mice perform this reversal learning task. The experimental setup requires minimal apparatus with no usage of external cues; thus, this setup is easy to equip under the microscope, including the two-photon microscope. In addition, the head-fixed experimental setup can use traditional or latest behavioral and physiological measurements, such as pupil size, eyelid size, facial expression, heart rate, and respiration. Using these techniques with this spatiotemporal Pavlovian head-fixed reversal learning task will pave the way for understanding behavioral flexibility.

In summary, we established a novel spatiotemporal Pavlovian head-fixed reversal learning task for mice. Licking responses on this Pavlovian head-fixed reversal learning task can be accurately quantified. Mice showed the hallmarks of the results of the reversal learning task in free-moving Pavlovian and operant conditioning. This novel head-fixed reversal learning task is a useful approach for studying the neurobiological mechanisms of behavioral flexibility as it can provide an informative framework for understanding the mechanism at the levels of genes, cells, neural circuits, behavior, and its computations.

## Methods

### Animals

We used adult C57BL/6J mice. Six mice (one male and five females) were used in the head-fixed reversal task. All animals were experimentally naive at the start of the experiment. Mice were maintained on a 12:12 light cycle. All experiments were conducted under the application of approximately 75 dB background white noise and during the dark phase of the animal’s light cycle. Mice were water deprived and received 10% sucrose solution during experiments. Their weights were monitored daily, with additional water provided after experimental training as needed. Mice had unrestricted access to food in their home cages. The experimental and housing protocols adhered to the Japanese National Regulations for Animal Welfare and were approved by the Animal Care and Use Committee of Keio University.

### Surgery

Mice were anesthetized with 1.0–2.5% isoflurane mixed with room air and placed in a stereotactic frame (942WOAE, David Kopf Instruments, Tujunga, CA). Then, a head post was implanted on the skull to allow mice to be head fixed during the experiment. Mice were housed individually without water-restriction as a recovery period for at least two weeks before the training began.

### Behavioral tasks

*Fixed-time schedule task* After recovery from surgery, mice were water-deprived in their home cage. On the first day of training, mice were head fixed and provided random water rewards to habituate them to the experimental environment. Behavioral experiments were conducted in a square behavioral chamber with a drinking steel spout in front of each animal’s mouth. Each mouse was kept on a covered elevated platform (custom-designed and 3D printed), with its head fixed by two stabilized clamps holding the sidebars of the head post. The heights of the tunnel and clamps were aligned prior to each session to ensure comfort. The spout and copper sheet under the stage were connected, and individual licking contacts between the mice and drinking needle were recorded using a contact touch sensor. Head-fixed mice were allowed to freely lick the spout. In the fixed-time schedule task, approximately 2 μL of 10% sucrose solution was delivered through the tube at 10-s intervals (Fig. 1). Sucrose delivery and recording of licking responses were executed using custom-made Python 3 (version 3.7.7) scripts with a custom-made relay circuit with solenoids. On each day of the experiment, we run one session that contains 250 trials. One trial consists of 10 s interval and the reward delivery.

*Fixed-time reversal learning task* After training on the fixed-time schedule task, a reversal learning task was initiated (Fig. 1). The experimental setup of the reversal learning task was identical to that of the fixed-time schedule task, except that two drinking steel spouts were placed in front of the mouth of mice. The distance between each licking spout was set at 4.5 mm. Sucrose solution was delivered through one of the two spouts. The rewarding spout was switched every 10 trials. The amount of sucrose solution delivered was calibrated to the same amount between each rewarding spout. We defined licking toward the rewarding spout as the correct response, and licking toward the non-rewarding spout as the error response. We defined anticipatory licking as licking from 2 to 0 s before reward delivery, and consummatory licking as licking from 0 to 2 s after reward delivery. We defined the error rates as the number of licking toward the non-rewarding spout divided by the total number of licking toward both spouts. The error rates in anticipatory licking are defined as the number of licking toward the non-rewarding spout divided by the total number of licking toward both spouts within 2 s before the reward delivery. The error rates in consummatory licking are defined as the number of licking toward the non-rewarding spout divided by the total number of licking toward both spouts within 2 s after the reward delivery. We defined the latencies of licking to the rewarding side of the spout by the timing from reward delivery to the first licking on that side.

### Analysis

RStudio 2022.02.1 + 461 (The R Foundation for Statistical Computing, Vienna, Austria) and GraphPad Prism 9.3.1 (GraphPad Software, La Jolla California USA) were used for analysis.

## Data Availability

The original codes written for the analysis are available from the corresponding author on request. Data supporting the findings of this study are available from the corresponding author upon reasonable request.
